# The Role of Ketogenic Diet and β-Hydroxybutyrate in the Prevention of Muscle Catabolism and Sarcopenia in Aging Populations: Mechanisms, Evidence, and Clinical Perspectives

**DOI:** 10.3390/nu18050761

**Published:** 2026-02-26

**Authors:** Claudia Venturini, Giulia Matacchione, Lucia Mancinelli, Sara Caccese, Michele Alfieri, Fabrizia Lattanzio, Fabiola Olivieri, Roberto Antonicelli

**Affiliations:** 1 Clinical Nutrition Unit, National Institute of Health and Science on Aging, IRCCS INRCA Ancona, 60127 Ancona, Italy; c.venturini@inrca.it; 2 Clinic of Laboratory and Precision Medicine, IRCCS INRCA, 60127 Ancona, Italy; 3 Cardiology Unit, IRCCS INRCA, 60127 Ancona, Italy; l.mancinelli@inrca.it (L.M.); m.alfieri@inrca.it (M.A.); r.antonicelli@inrca.it (R.A.); 4 Advanced Technology Center for Aging Research and Geriatric Mouse Clinic, IRCCS INRCA, 60121 Ancona, Italy; s.caccese@inrca.it (S.C.); f.olivieri@inrca.it (F.O.); 5 Department of Biomedical Sciences and Public Health, Marche Polytechnic University, Marche University Hospital, 60126 Ancona, Italy; 6 Scientific Direction, IRCCS INRCA Ancona, 60124 Ancona, Italy; direzionescientifica@inrca.it; 7 Department of Clinical and Molecular Sciences (DISCLIMO), Università Politecnica delle Marche, 60126 Ancona, Italy

**Keywords:** ketogenic diet, sarcopenia, β-hydroxybutyrate, muscle mass, aging

## Abstract

Sarcopenia, characterized by the progressive loss of skeletal muscle mass, strength, and function, represents a growing public health challenge in aging populations. Emerging mechanistic evidence suggests that ketogenic diets (KDs) and elevated circulating β-hydroxybutyrate (βOHB) levels may offer selective and context-dependent nutritional strategies to support muscle health during aging. This review summarizes current evidence on the effects of ketogenic diets and ketone body metabolism on muscle mass and function, with a focus on underlying molecular mechanisms and clinical relevance in older adults. βOHB acts not only as an alternative energy substrate but also as a signaling molecule, notably through histone deacetylase inhibition and modulation of inflammatory pathways. Nutritional ketosis in humans typically results in circulating βOHB concentrations of approximately 0.5–3.0 mM, which may be sufficient to engage some of these signaling pathways, although the extent of these effects in human tissues remains incompletely defined. Preclinical studies indicate that long-term ketogenic diets preserve muscle mass, strength, and mitochondrial function in aging models. Limited clinical evidence, largely derived from populations with sarcopenic obesity or metabolic comorbidities, suggests that protein-adequate ketogenic diets, when implemented as an adjunct to physical exercise, may help preserve fat-free mass and improve functional outcomes, while exogenous ketones show potential to augment post-exercise anabolic signaling. Overall, the integration of mechanistic and preliminary clinical data provides a supplementary and exploratory framework suggesting that ketogenic diets may represent a promising adjunctive strategy for sarcopenia prevention, although well-designed long-term randomized controlled trials are required to define their efficacy, safety, and optimal clinical application.

## 1. Introduction

Sarcopenia is defined by the European Working Group on Sarcopenia in Older People (EWGSOP2) as a “muscle disease characterized by progressive loss of muscle mass, strength, and function” [1,2]. This condition significantly increases the risk of falls, disability, institutionalization, and mortality, thus imposing substantial healthcare costs [3]. The pathophysiology of sarcopenia involves complex interactions between aging-related factors, including chronic inflammation, mitochondrial dysfunction, hormonal changes, reduced physical activity, and nutritional inadequacy [4]. Traditional therapeutic approaches have focused primarily on resistance exercise training and protein supplementation, with limited pharmacological options available [5]. However, these interventions often provide only modest benefits, highlighting the need for novel therapeutic strategies.

Ketogenic diets (KDs), characterized by high fat (70–80%), moderate protein (15–25%), and very low carbohydrate (<10%) content, induce a metabolic state of ketosis where ketone bodies—particularly β-hydroxybutyrate (βOHB)—serve as alternative fuel sources [6]. Beyond their role as metabolic substrates, ketone bodies have emerged as important signaling molecules with pleiotropic effects on cellular function, gene expression, and stress resistance [7].

Mechanistic insights have revealed that βOHB functions as an endogenous histone deacetylase (HDAC) inhibitor, modulating epigenetic regulation and enhancing expression of genes involved in oxidative stress resistance and mitochondrial biogenesis [8,9]. These properties, combined with the metabolic flexibility provided by ketosis, suggest potential benefits for maintaining muscle health during aging.

Recent preclinical evidence further strengthens the rationale for investigating ketogenic dietary strategies in the context of musculoskeletal aging. In aged murine models, long-term ketogenic interventions have been shown to preserve skeletal muscle mass and function, improve grip strength and functional performance, and enhance mitochondrial oxidative capacity and bioenergetic efficiency in skeletal muscle [10,11,12]. Additional experimental work suggests that ketogenic interventions may confer resilience to age- and disease-related neuromuscular and metabolic stress, supporting the functional impact of ketosis on pathways relevant to sarcopenia [13]. In parallel, several recent studies and mechanistic reviews have emphasized the pleiotropic roles of ketone bodies in metabolism, signaling, and aging biology, framing ketogenic interventions as potential modulators of health span and age-related functional decline [6,7,14,15]. Together, these advances provide a timely and mechanistically grounded rationale for a focused evaluation of ketogenic strategies in the context of sarcopenia.

This review examines the current state of evidence regarding ketogenic diets and ketone body metabolism in the context of sarcopenia prevention and management in older adults, with emphasis on molecular mechanisms, preclinical findings, and emerging clinical applications across diverse aging populations.

## 2. Biological Mechanisms Linking β-Hydroxybutyrate to Muscle Preservation

In skeletal muscle, ketogenic diets induce a range of adaptations that may mitigate age-related muscle deterioration. These dietary interventions enhance metabolic flexibility by increasing the capacity for fatty acid and ketone body oxidation, thereby reducing reliance on glucose metabolism while maintaining efficient ATP production [11]. Such flexibility may be especially advantageous during periods of reduced energy intake or metabolic stress, conditions that commonly occur with aging. In addition, evidence from preclinical models indicates that long-term ketogenic diets preferentially preserve oxidative type I and type IIa muscle fibers while promoting a shift away from glycolytic type IIb fibers, a redistribution associated with greater fatigue resistance and improved metabolic health [10]. Furthermore, exogenous ketone supplementation has been shown to potentiate activation of the mTORC1 signaling pathway following resistance exercise, suggesting a potential enhancement of the anabolic response to muscle-building stimuli and improved support for muscle protein synthesis [16].

**Figure 1:**
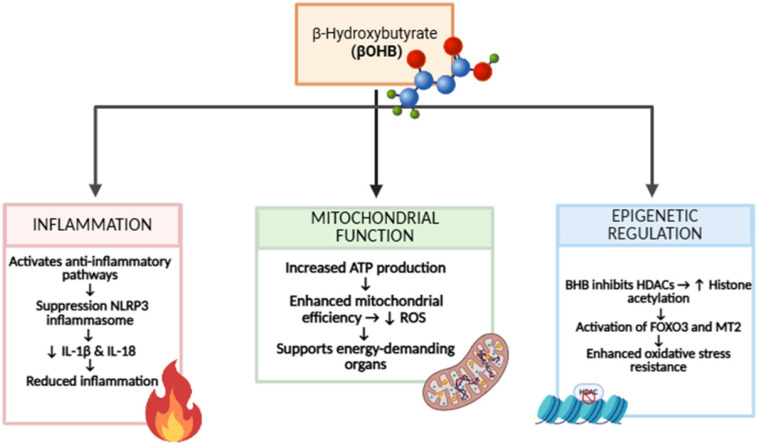
Schematic overview of the pleiotropic effects of β-hydroxybutyrate (βOHB) on inflammation, mitochondrial function, and epigenetic regulation. βOHB exerts anti-inflammatory actions in part through suppression of NLRP3 inflammasome activation, leading to reduced production of pro-inflammatory cytokines such as IL-1β and IL-18. At the mitochondrial level, βOHB supports cellular bioenergetics by enhancing ATP production and mitochondrial efficiency while limiting excessive reactive oxygen species (ROS) generation, thereby supporting energy-demanding tissues. In parallel, βOHB acts as an epigenetic signaling metabolite by inhibiting histone deacetylases (HDACs), promoting histone acetylation and the activation of stress-resistance pathways (e.g., FOXO3 and MT2), ultimately contributing to improved oxidative stress resilience and metabolic adaptation.

Such observations highlight the emerging view that βOHB exerts pleiotropic effects on skeletal muscle that extend beyond energy provision (Figure 1).

Indeed, βOHB serves dual roles as both an energy substrate and a regulatory molecule. As demonstrated by Shimazu et al., βOHB acts as an endogenous and specific inhibitor of class I histone deacetylases (HDACs 1, 3, and 4) [8,9]. This HDAC inhibition leads to increased histone acetylation, particularly at H3K9 and H3K14 residues, resulting in enhanced transcription of genes involved in cellular stress resistance and coordination of a set of mechanistic effects that support cellular and metabolic adaptation. Particularly, HDAC inhibition mediated by βOHB enhances antioxidant capacity via upregulation of FOXO3A and metallothionein-2 (MT2), thereby improving cellular resistance to oxidative stress [8].

Recent evidence indicates that βOHB exerts pleiotropic epigenetic and transcriptional effects that extend beyond its role as an energy substrate. In particular, histone H3 lysine 9 β-hydroxybutyrylation (H3K9bhb) has been identified as a novel epigenetic mechanism underlying the protective effects of βOHB in limiting kidney injury and reducing hypertension [17]. In parallel, βOHB selectively suppresses histone deacetylase 2 (HDAC2), disrupts its binding to the *Sirt7* promoter, and promotes SIRT7 activation, thereby modulating mitochondrial biogenesis [18]. Moreover, βOHB inhibits histone deacetylase 3 (HDAC3) and enhances H3K14 acetylation at the *Claudin-5* promoter, leading to increased claudin-5 expression and attenuation of diabetes-associated cardiac microvascular hyperpermeability [19]. Concurrently, βOHB promotes mitochondrial biogenesis through activation of PGC-1α and associated transcriptional programs, supporting mitochondrial quality and function [20,21]. In particular, βOHB increases the AMP/ATP ratio and/or directly activates AMP-activated protein kinase (AMPK), leading to phosphorylation of AMPK at Thr172 [22]. Activated AMPK, in turn, directly phosphorylates PGC-1α at Thr177 and Ser538, thereby enhancing its stability and transcriptional activity [23]. Moreover, βOHB acts as a calorie restriction mimetic by increasing the intracellular NAD^+^/NADH ratio [24], which promotes activation of the NAD^+^-dependent deacetylase SIRT1; SIRT1-mediated deacetylation of PGC-1α further enhances its transcriptional activity and protein stability [25]. Collectively, these convergent AMPK- and SIRT1-dependent pathways place PGC-1α as a central downstream effector of βOHB signaling, linking ketone body metabolism to mitochondrial biogenesis and oxidative metabolic reprogramming [26]. However, these mechanistic studies were conducted primarily in cell culture and animal models, and the relevance of these pathways at physiological ketone concentrations achieved through dietary intervention (typically 0.5–3 mM) in humans remains to be fully established.

Ketogenic diets and the resulting state of ketosis may also counteract the chronic low-grade inflammation, commonly named inflammaging, that is a major contributor to the development and progression of sarcopenia [27]. Notably, βOHB has been shown to directly inhibit activation of the NLRP3 inflammasome, leading to reduced production of pro-inflammatory cytokines such as IL-1β and TNF-α [28,29].

In conclusion, extracellular and/or intracellular acidification enables βOHB and related short-chain carboxylic acids (SCCAs) to inhibit NLRP3 inflammasome activation, at least in part via GPR41/FFAR3 signaling, thereby broadening the spectrum of metabolites capable of modulating this key pro-inflammatory pathway during states of energetic stress and creating a permissive context for the therapeutic exploitation of ketone bodies as anti-inflammatory agents [30].

In parallel, ketogenic diets appear to alleviate cellular stress by reducing markers of endoplasmic reticulum (ER) stress, including decreased activation of PERK, IRE1, and eIF2α signaling pathways, which are commonly upregulated in aging and metabolic dysfunction [13]. Additionally, lower circulating glucose levels combined with enhanced antioxidant defenses may limit the formation of advanced glycation end products (AGEs), thereby attenuating AGE-associated inflammatory signaling and tissue damage [31]. Collectively, these anti-inflammatory and stress-modulating effects may contribute to preservation of muscle integrity and function during aging.

## 3. Preclinical Evidence

Several landmark preclinical studies have provided compelling evidence for the muscle-preserving effects of ketogenic diets in aging animal models. Wallace et al. conducted a comprehensive investigation in C57BL/6 mice, comparing lifelong ketogenic diet feeding with a control diet and demonstrating significant preservation of hind limb muscle mass at 26 months of age, along with maintenance of muscle fiber cross-sectional area and reduced intramuscular fat accumulation [10]. These structural benefits were accompanied by a shift toward more oxidative muscle fiber types (type I and IIa), increased mitochondrial content, and enhanced expression of genes involved in mitochondrial biogenesis and antioxidant defense, indicating improved muscle metabolic quality. More recent studies have extended these observations to functional outcomes, showing that ketogenic diets preserve grip strength and motor unit number in aged mice, thereby suggesting protective effects on both muscle mass and neuromuscular integrity [12].

The impact of ketogenic diets was also examined in mice with type 2 diabetes, a condition commonly associated with accelerated sarcopenia, especially in older patients [13,32]. Their findings revealed preservation of muscle mass and strength, increased muscle fiber cross-sectional area, and improved grip strength in ketogenic diet-fed diabetic mice, together with reduced expression of NLRP3 inflammasome components and endoplasmic reticulum stress markers. These adaptations were accompanied by enhanced insulin sensitivity and improved glucose homeostasis, underscoring the potential of ketogenic diets to confer particular benefits in populations with metabolic comorbidities that predispose to muscle loss.

Several interconnected mechanisms may underlie the muscle-protective effects of ketogenic diets. Long-term ketogenic diet feeding has been shown to preserve mitochondrial mass and respiratory capacity in aging skeletal muscle, potentially mediated by enhanced PGC-1α signaling and a reduction in oxidative damage, thereby supporting mitochondrial quality and energy production [10]. In addition, ketogenic diets appear to promote protein homeostasis by attenuating overall protein turnover; this effect is achieved through suppression of major proteolytic pathways, including the ubiquitin–proteasome system and autophagy, as well as modulation of translation initiation, resulting in net preservation of muscle protein content [13]. Emerging evidence also suggests that ketogenic diets may help maintain neuromuscular junction integrity during aging, which could contribute to preserved muscle function, although this mechanism remains less well characterized and warrants further investigation [12].

## 4. Clinical Evidence in Human Populations

Translating mechanistic and preclinical findings into clinical practice requires careful evaluation of human evidence, particularly in heterogeneous aging populations. Importantly, mechanistic validity does not necessarily equate to proven clinical efficacy, and the strength of evidence differs substantially between preclinical and human studies. To date, clinical studies investigating ketogenic dietary strategies in older adults remain limited in number and heterogeneous in design, and the available evidence is largely confined to populations with sarcopenic obesity and/or metabolic comorbidities (e.g., obesity, insulin resistance, type 2 diabetes), rather than to older adults with primary, non-obesity-related sarcopenia. As such, the generalizability of these findings to the broader population of older adults with sarcopenia remains uncertain and should be interpreted with caution. Sarcopenic obesity, defined by the coexistence of reduced muscle mass and excess adiposity, represents a particularly complex clinical phenotype associated with compounded metabolic dysfunction and heightened functional impairment [33]. Within this context, several clinical investigations have evaluated ketogenic dietary strategies. In a small pilot randomized controlled trial comparing a very low-calorie ketogenic diet (VLCKD) alone with VLCKD combined with interval training in adults with sarcopenic obesity, the combined intervention resulted in less fat-free mass loss compared with VLCKD alone over 6 weeks [34]. However, both groups experienced reductions in fat-free mass, and the clinical significance of this difference over such a short intervention period remains uncertain. The combined intervention was also associated with greater preservation of fat-free mass, significant improvements in Short Physical Performance Battery (SPPB) scores, and marked reductions in inflammatory markers, including C-reactive protein and IL-6, while demonstrating acceptable tolerability and short-term safety. Complementary clinical observations from European centers further suggest that protein-adequate ketogenic diets (approximately 1.2–1.5 g/kg/day of protein), when combined with resistance exercise, may optimize the balance between lean mass preservation and fat loss in older individuals with sarcopenic obesity [35]. Nevertheless, these observations remain preliminary and derive from small cohorts and short-term interventions. Given the challenges of long-term adherence to strict ketogenic diets, the use of exogenous ketones has been explored primarily in metabolically compromised or physically active populations, rather than in individuals with established sarcopenia per se. Vandoorne et al. demonstrated that ketone ester ingestion following resistance exercise enhanced mTORC1 signaling in human skeletal muscle, as evidenced by increased phosphorylation of downstream targets such as S6K1 and 4E-BP1, leading to elevated muscle protein synthesis rates without impairing glycogen resynthesis [16]. More recent trials indicate that ketone ester supplementation can acutely raise circulating βOHB concentrations to therapeutic levels (1–3 mM) in older adults, with good tolerability and potential to augment post-exercise anabolic signaling [36]. However, whether these acute molecular responses translate into sustained improvements in muscle mass or functional outcomes in sarcopenic older adults remains unknown. In parallel, alternative dietary models aimed at improving adherence have been explored in high-risk older adults with metabolic disease. A Mediterranean ketogenic nutrition program incorporating olive oil, nuts, fish, and low-carbohydrate vegetables while maintaining ketosis demonstrated preliminary improvements in adherence compared with traditional ketogenic diets, while preserving metabolic benefits [37]. Collectively, these human studies provide preliminary support for the translational relevance of the proposed mechanisms; however, they do not yet establish clinical efficacy, underscoring the need for adequately powered, long-term randomized controlled trials with clinically meaningful endpoints.

Importantly, the potential applicability of such modified ketogenic approaches to older adults with sarcopenia in the absence of metabolic disease has yet to be systematically evaluated.

## 5. Safety Considerations in Older Populations

Clinical evidence accumulated over recent years suggests that ketogenic diets are generally well tolerated in older populations when carefully planned, individualized, and clinically supervised, although several safety considerations must be taken into account. Available data do not support concerns regarding accelerated renal function decline, as studies in older populations report stable or modestly improved renal parameters when protein intake and hydration are adequately managed [34]. Cardiometabolic effects are largely favorable, including reductions in blood pressure and triglycerides and increases in HDL cholesterol, while responses in LDL cholesterol are more variable, highlighting the need for individualized lipid monitoring and attention to dietary fat quality [38]. Transient gastrointestinal and systemic symptoms may occur during the initial adaptation to ketosis but can usually be mitigated through gradual carbohydrate reduction, adequate fluid and electrolyte intake, and clinical support [39]. From a practical perspective, ketogenic interventions in older adults should be tailored to functional status, comorbidity burden, and living environment, with particular emphasis on maintaining adequate protein intake (generally 1.2–1.5 g/kg/day) and micronutrient sufficiency. Importantly, ketogenic diets should be integrated with structured resistance exercise, which remains a cornerstone of sarcopenia prevention. In frailer or institutionalized older individuals, more flexible ketogenic approaches allowing higher carbohydrate intakes (approximately 30–50 g/day) may improve feasibility and adherence, with close monitoring of nutritional status and physical function. In cases where dietary adherence is challenging or caloric intake is insufficient, supplementation with exogenous ketones may represent a practical alternative to induce mild ketosis without imposing strict dietary restrictions. Finally, the presence of comorbid conditions necessitates additional caution and individualized management. In older individuals with type 2 diabetes, ketogenic diets appear particularly promising due to their potent glucose-lowering effects and potential to ameliorate diabetes-associated muscle dysfunction; however, close monitoring of blood glucose and proactive adjustment of antidiabetic medications are mandatory to avoid hypoglycemia [13]. In those with cardiovascular disease, ketogenic diets are generally safe but should be approached cautiously in patients with advanced heart failure or those receiving complex pharmacological regimens, where fluid balance and electrolyte status may be more labile [40]. Emerging evidence also suggests that ketosis may confer cognitive benefits in older adults with mild cognitive impairment or neurodegenerative conditions, making this population an attractive target for future research, provided that nutritional adequacy and overall safety are carefully maintained [41].

## 6. Clinical Implementation Framework

The clinical application of ketogenic diets in older populations requires a structured and multidisciplinary framework to maximize efficacy while ensuring safety. Given the heterogeneity of aging individuals in terms of functional status, metabolic health, and comorbidities, ketogenic interventions should be conducted through assessment, followed by phased implementation and ongoing monitoring.

### 6.1. Assessment and Screening

Prior to initiating a ketogenic diet in older adults, a comprehensive baseline assessment is essential to identify sarcopenia severity, metabolic risk factors, and potential contraindications. Screening for sarcopenia should include objective measures of muscle strength, such as handgrip dynamometry, alongside functional assessments including the Short Physical Performance Battery (SPPB) and gait speed evaluation. Where available, quantitative assessment of muscle mass using dual-energy X-ray absorptiometry (DXA) or validated bioelectrical impedance analysis should be performed to establish baseline appendicular lean mass and monitor longitudinal changes [1]. Metabolic evaluation should encompass a complete metabolic panel, fasting glucose and insulin, HbA1c, lipid profile, kidney and liver function tests, and thyroid function, allowing for identification of underlying metabolic disorders and establishment of reference values for follow-up. A detailed medication review is particularly critical in older individuals, with special attention to glucose-lowering agents, antihypertensive medications, and anticoagulants, as ketogenic diets may necessitate dose adjustments to avoid hypoglycemia, hypotension, or bleeding risk. Finally, a structured nutritional assessment should evaluate habitual dietary intake, appetite, swallowing function, gastrointestinal tolerance, and food preferences, enabling individualized meal planning and improving adherence.

### 6.2. Implementation Protocol

Ketogenic diet implementation in older populations is best approached through a phased protocol that allows gradual metabolic adaptation, minimizes adverse effects, and supports long-term sustainability. A phased approach may facilitate the safe and sustainable implementation of ketogenic diets in older adults, as reported in Table 1. To summarize, an initial adaptation phase involves a gradual reduction in carbohydrate intake to induce ketosis while minimizing early adverse symptoms, with emphasis on adequate protein intake, inclusion of unsaturated fat sources, and attention to electrolyte balance. This is followed by an optimization phase aimed at achieving and maintaining nutritional ketosis, during which macronutrient composition is individualized and resistance exercise is introduced or intensified to support muscle mass and functional outcomes. Finally, a maintenance phase focuses on long-term adherence and lifestyle integration, with periodic reassessment of nutritional status, metabolic health, and physical performance and, where appropriate, the use of intermittent or cyclical ketogenic strategies to enhance feasibility.

### 6.3. Monitoring and Outcomes

Clinical monitoring should include clearly defined outcome measures. Primary outcomes include changes in muscle mass (DXA-derived appendicular lean mass), muscle strength assessed by handgrip dynamometry, and physical performance metrics such as SPPB scores and gait speed. Secondary outcomes should encompass overall body composition, metabolic markers (fasting glucose, insulin, lipid profile), inflammatory biomarkers (CRP, IL-6), and patient-reported quality-of-life measures. Safety monitoring remains a priority throughout the intervention and should include periodic assessment of renal and liver function, electrolyte balance, and blood pressure, particularly during the early adaptation phase and in individuals with comorbidities.

## 7. Exogenous β-Hydroxybutyrate Versus Nutritional Ketosis

### 7.1. Mechanistic and Metabolic Distinctions

Several authors have proposed exogenous βOHB supplementation as an alternative strategy to induce ketosis without strict carbohydrate restriction; however, it is critical to distinguish transient elevations in circulating ketones from the coordinated systemic metabolic adaptations elicited by a ketogenic diet [42,43,44]. Ketone esters, particularly monoesters of (R)- βOHB, can acutely raise blood βOHB concentrations in humans to approximately 2–3 mmol/L, whereas ketone salts typically produce more modest ketonemia and are limited by gastrointestinal tolerability and mineral load [44,45,46,47]. Importantly, all exogenous formulations increase circulating ketones independently of hepatic ketogenesis, thereby bypassing the endocrine, enzymatic, and transcriptional adaptations that characterize nutritional ketosis [44,45,46,47,48]. Controlled human studies consistently show that acute βHB ingestion elevates ketonemia, lowers blood glucose, and suppresses circulating non-esterified fatty acids, reflecting inhibition of adipose tissue lipolysis; these effects contrast with the metabolic phenotype induced by carbohydrate restriction, which is characterized by enhanced lipolysis and fatty acid oxidation [49,50,51,52]. While preclinical studies suggest that ketone ester administration may influence mitochondrial substrate utilization and oxidative metabolism, human data indicate that these effects are primarily acute, and direct evidence for sustained improvements in mitochondrial biogenesis or mitochondrial function attributable to βOHB supplementation alone remains scarce [47,53,54].

### 7.2. Clinical Implications and Translational Relevance

Evidence regarding longer-term βOHB supplementation remains limited and does not support exogenous ketones as a substitute for a ketogenic diet in achieving durable metabolic remodeling [55,56]. Sustained supplementation rarely maintains ketone concentrations comparable to those observed during nutritional ketosis, and consistent improvements in body weight, glycemic control, or insulin sensitivity have not been demonstrated independently of dietary intervention. Based on current evidence, exogenous βHB supplementation should therefore be considered an adjunctive or experimental strategy, rather than a replacement for a ketogenic diet [57]. Its potential utility may lie in inducing short-term, targeted ketonemia in specific clinical or research contexts where dietary carbohydrate restriction is not feasible. However, for outcomes such as weight loss, visceral fat reduction, improvements in insulin sensitivity, and long-term cardiometabolic risk reduction, overall dietary pattern and sustained metabolic adaptation remain the primary determinants [42,43,44]. In the context of sarcopenia and age-related muscle loss, these limitations are particularly relevant, as older adults require sustained metabolic and anabolic stimuli—most notably adequate protein intake and resistance exercise—to preserve muscle mass and function. Transient ketonemia induced by exogenous βOHB, in the absence of broader dietary and lifestyle adaptations, is therefore unlikely to provide sufficient or durable protection against the multifactorial drivers of muscle catabolism in aging populations.

## 8. Practical Recommendations

### 8.1. Macronutrient Targets for Older Populations

Protein intake represents a critical determinant of success when implementing ketogenic diets in older adults. Daily protein intake should generally range from 1.2 to 1.5 g/kg body weight, with higher intakes of up to 2.0 g/kg potentially beneficial in frail individuals or those with pronounced anabolic resistance [58]. High biological value protein sources should be emphasized, with distribution across meals to provide approximately 25–30 g of protein per meal. Inclusion of 2.5–3 g of leucine per meal is recommended to maximize stimulation of muscle protein synthesis. Carbohydrate intake should typically be maintained between 20 and 50 g/day [38], individualized according to ketone response, metabolic health, and tolerance [58]. Priority should be given to nutrient-dense, low-glycemic vegetables and adequate fiber intake to support gastrointestinal health. Dietary fat should account for approximately 70–80% of total caloric intake, with emphasis on monounsaturated and omega-3 fatty acids. Supplementation with medium-chain triglycerides (MCTs), in doses of 10–20 g/day, may further enhance endogenous ketone production [59].

### 8.2. Supplementation and Exercise Integration

Electrolyte supplementation is foundational during ketogenic interventions in older populations, with recommended daily intakes of approximately 2–3 g sodium, 3–4 g potassium, and 400–600 mg magnesium. Vitamin D supplementation (1000–2000 IU/day, adjusted based on serum levels), omega-3 fatty acids (1–2 g/day of EPA/DHA), and a comprehensive multivitamin-mineral supplement are commonly indicated to address potential micronutrient gaps. Optional adjuncts include exogenous ketones (10–15 g post-exercise) to enhance anabolic signaling, creatine monohydrate (3–5 g/day) for additional muscle and cognitive benefits, and probiotics to support gut health during dietary transition [60,61]. Resistance exercise remains a cornerstone of sarcopenia prevention and should be systematically integrated with ketogenic diet interventions. Training should be performed two to three times per week on non-consecutive days, using intensities corresponding to approximately 70–85% of one-repetition maximum for 6–12 repetitions per set. Progressive overload through gradual increases in resistance, volume, or training density is essential to sustain adaptations. Exercise programs should prioritize major muscle groups and functional movement patterns relevant to daily activities. When appropriate, exogenous ketone supplementation within 30–60 min post-exercise may further support recovery and anabolic signaling [60].

## 9. Limitations and Future Directions

Despite growing interest in ketogenic diets as a potential strategy to mitigate sarcopenia and associated metabolic dysfunction in older adults, the current evidence base is characterized by several important limitations that constrain both interpretation and clinical translation. It should be noted that the majority of evidence supporting the HDAC inhibitory and anti-inflammatory effects of β-hydroxybutyrate (βOHB) derives from in vitro experiments and animal models, often employing βOHB concentrations that exceed those typically achieved in humans through dietary ketosis or exogenous ketone supplementation. In humans, nutritional ketosis induced by ketogenic diets generally results in circulating βOHB concentrations in the range of ~0.5–3.0 mM, whereas many mechanistic studies have used higher or supraphysiological concentrations. Moreover, most available clinical studies are short in duration and involve relatively small sample sizes, limiting statistical power and precluding firm conclusions regarding long-term sustainability, adherence, safety, and clinically meaningful effects on muscle mass, strength, and physical function in a condition that evolves over many years. Furthermore, substantial heterogeneity across studies in participant characteristics, dietary protocols, intervention durations, and outcome measures complicates cross-study comparisons and limits the feasibility of high-quality meta-analyses. Beyond methodological constraints, significant barriers to real-world implementation remain, including limited familiarity and formal training in ketogenic dietary approaches among healthcare providers, the nutritional complexity of maintaining adequate macronutrient balance while preventing micronutrient deficiencies, potential cost-related barriers, and social, cultural, and functional factors that may compromise long-term adherence in older adults. Addressing these gaps will require well-designed, large-scale, multicenter randomized controlled trials with extended intervention periods (12–24 months) to evaluate long-term efficacy, safety, and comparative effectiveness relative to more conventional dietary strategies, such as high-protein, moderate-carbohydrate diets. Future research should also systematically characterize long-term safety across heterogeneous older populations with varying degrees of frailty and comorbidity and advance precision nutrition approaches that move beyond one-size-fits-all prescriptions. In this context, the integration of wearable technologies for continuous monitoring of ketosis, physical activity, and physiological responses, alongside artificial intelligence-driven algorithms for dynamic and individualized dietary adjustment, may enhance adherence and early detection of adverse effects. Finally, complementary mechanistic studies are needed to elucidate the biological pathways through which ketosis and βOHB may exert muscle-protective effects, thereby informing the development of more effective, scalable, and personalized strategies for the prevention and management of sarcopenia in aging populations.

## 10. Conclusions

The convergence of mechanistic insights and emerging clinical evidence positions ketogenic diets and ketone body metabolism as potentially promising strategies for the prevention and management of sarcopenia in aging populations. Central to this rationale is the dual role of βOHB as both an alternative energy substrate and an epigenetic signaling molecule. By functioning as an endogenous HDAC inhibitor, βOHB promotes cellular stress resistance, mitochondrial biogenesis, and anti-inflammatory signaling—processes directly relevant to the pathophysiology of age-related muscle loss. Preclinical studies provide robust support for these mechanisms, as long-term ketogenic diet interventions in animal models consistently demonstrate preservation of muscle mass, improvements in mitochondrial function, enhanced oxidative capacity, and increased resilience to metabolic and oxidative stress. Complementing these findings, early clinical trials, particularly among populations with sarcopenic obesity, indicate that ketogenic diets—when combined with resistance exercise—may help maintain lean mass, improve functional performance, and reduce inflammatory markers. The use of exogenous ketones further expands the therapeutic landscape by enabling transient ketosis and associated anabolic and metabolic signaling without necessitating strict dietary restrictions, potentially offering a more practical option for selected older individuals. Nevertheless, the framework presented here should be regarded as supplementary and exploratory in nature, as the current evidence base is still limited and largely derived from preclinical studies and short-term clinical trials.

Despite these promising signals, several caveats warrant consideration. Effective implementation of ketogenic interventions in older adults requires adequate protein intake, integration with resistance exercise, and careful monitoring of safety parameters, including renal function, electrolyte balance, and medication adjustments. Substantial interindividual variability in response underscores the need for personalized approaches and ongoing assessment. Moreover, the evidence base remains constrained by short trial durations, small sample sizes, and heterogeneous study designs, highlighting the need for larger, long-term randomized controlled trials. Taken together, the available evidence supports a coherent mechanistic framework linking ketosis and β-hydroxybutyrate signaling to pathways implicated in muscle aging; however, clinical efficacy in terms of sarcopenia prevention or reversal has not yet been conclusively demonstrated and remains to be established by dedicated long-term clinical trials. Practical considerations, including provider education, access to nutritional support, and adherence strategies, will be critical for real-world implementation. While ketogenic diets are not a panacea for sarcopenia, they may represent a valuable adjunct to comprehensive lifestyle interventions aimed at preserving muscle mass, function, and metabolic health. With careful clinical oversight, individualized planning, and integrated exercise and nutritional strategies, ketogenic diets could offer selected older individuals a novel supportive tool to maintain muscle integrity and functional independence throughout the aging process.

## Figures and Tables

**Table 1:** Phased implementation protocol for ketogenic diets in older populations, summarizing duration, macronutrient targets, monitoring strategies, and clinical goals for each phase.

Phase	Time- Frame	Dietary Targets	Monitoring Parameters	Clinical Goals
Phase 1: Adaptation	Weeks 1–2	Gradual reduction in carbohydrate intake to <30 g/day; emphasis on high-quality protein sources (fish, poultry, eggs, and dairy); liberal use of healthy fats (olive oil, nuts, seeds, and avocado); initiation of electrolyte supplementation (sodium, potassium, and magnesium)	Daily or frequent monitoring of ketones (blood or urine); assessment of hydration status and electrolyte balance; monitoring of gastrointestinal tolerance, fatigue, and orthostatic symptoms	Facilitate metabolic adaptation to ketosis; minimize adverse symptoms during transition; maintain protein adequacy and prevent early lean mass loss
Phase 2: Optimization	Weeks 3–8	Achievement and stabilization of nutritional ketosis (β-hydroxybutyrate 0.5–1.5 mM); individualized adjustment of macronutrient ratios; continued emphasis on protein adequacy (1.2–1.5 g/kg/day); integration of resistance exercise training	Periodic assessment of body composition (DXA or BIA where available); evaluation of muscle strength (grip strength); functional performance (SPPB and gait speed); metabolic markers (glucose, insulin, and lipids)	Optimize fat loss while preserving or improving lean mass; enhance muscle strength and functional capacity; support metabolic health
Phase 3: Maintenance	Month 3 and beyond	Long-term dietary adherence strategies; potential transition to intermittent or cyclical ketogenic approaches; continued protein sufficiency; flexible carbohydrate targets based on individual tolerance and goals	Periodic reassessment of nutritional status, renal and liver function, lipid profile, inflammatory markers, and physical performance; monitoring of adherence and quality of life	Sustain metabolic and musculoskeletal benefits; enhance long-term feasibility and lifestyle integration; prevent relapse of sarcopenia and metabolic dysfunction

## Data Availability

No new data were created or analyzed in this study. Data sharing is not applicable to this article.
